# Multiple diagnostic tests demonstrate an increased risk of canine heartworm disease in northern Queensland, Australia

**DOI:** 10.1186/s13071-021-04896-y

**Published:** 2021-08-09

**Authors:** Jessica L. Panetta, Nichola Eliza Davies Calvani, Bronwyn Orr, Aldo Gianfranco Nicoletti, Michael P. Ward, Jan Šlapeta

**Affiliations:** Sydney School of Veterinary Science, Faculty of Science, The University of Sydney, Sydney, NSW 2006 Australia

**Keywords:** *Dirofilaria immitis*, Prevalence, Microfilariae, Shelter dogs, Antigen test, Heat-treatment, Knott’s test, PCR, Queensland, Australia

## Abstract

**Background:**

Canine heartworm (*Dirofilaria immitis*) is a life-threatening infection of dogs with a global distribution. Information on the prevalence of *D*. *immitis* and associated risk factors for canine heartworm antigen positivity—and thus disease—in Australia is scarce or outdated. The current reference method for *D*. *immitis* diagnosis in dogs is* via* the detection of heartworm antigen in blood using commercially available microwell-based enzyme-linked immunosorbent assays (ELISAs). Heat treatment of canine plasma prior to testing has been suggested to increase test sensitivity. The aim of the current study was to estimate the prevalence of *D*. *immitis* in dogs confined to shelters in Queensland, Australia. The impact of heat treatment on antigen test results was also assessed.

**Methods:**

Blood samples (*n* = 166) were collected directly from dogs in seven shelters across Queensland (latitudinal span of approx. 1700 km) into EDTA blood collection tubes. A commercially available ELISA (DiroCHEK®) was used to detect canine heartworm antigen in untreated and heat-treated plasma. Whole blood was concurrently tested for the presence of microfilariae and *D*. *immitis* DNA using a modified Knott’s test and real-time PCR, respectively. Risk factors (age, gender, source, location) associated with the odds of positivity for canine heartworm were assessed using binary logistic regression models.

**Results:**

A total of 16 dogs (9.6%; 95% confidence interval [CI]: 5.9–15.2%) were positive for canine heartworm based on combined test results. Heat treatment did not impact on the positivity of *D*. *immitis* antigen within samples (Cohen’s kappa = 0.98), but the optical density was significantly increased in paired plasma samples for *D*. *immitis* antigen-positive samples (Wilcoxon matched-pairs signed rank test, two-tailed *P* < 0.01). Location of the dog in a shelter in northern Queensland was the only risk factor significantly associated with the odds of a dog being more likely to be *D*. *immitis* antigen positive (odds ratio: 4.39; 95% CI: 1.26–13.51). All samples positive for the modified Knott’s test were also positive for *D*. *immitis* DNA by PCR.

**Conclusions:**

This study demonstrated the presence of heartworm-positive dogs in shelters in Queensland, with positive animals significantly more likely to occur in northern Queensland than southern Queensland. Sustained testing for the presence of *D*. *immitis* microfilariae and antigen remain important diagnostic tools in areas with known and re-emerging canine heartworm activity.

**Graphical Abstract:**

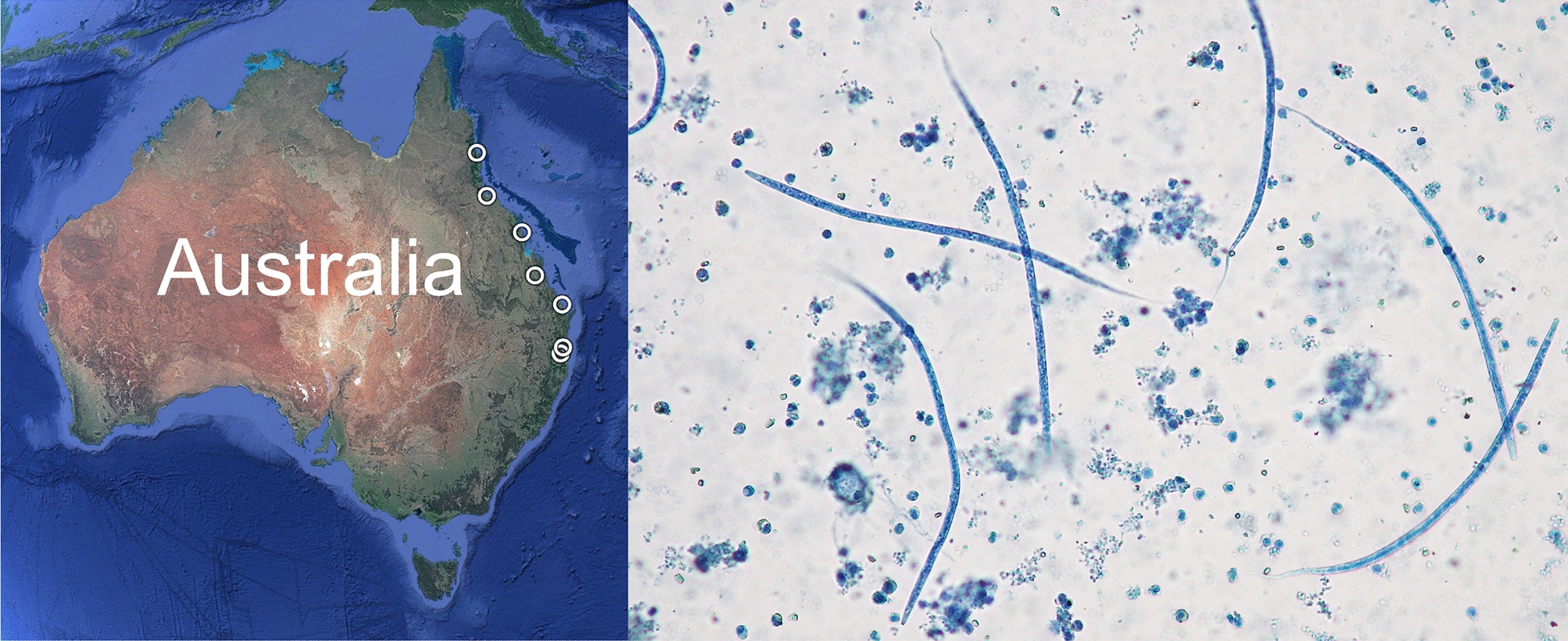

**Supplementary Information:**

The online version contains supplementary material available at 10.1186/s13071-021-04896-y.

## Background

Heartworm disease, caused by the parasitic nematode *Dirofilaria immitis*, is a life-threatening disease affecting canines throughout the world [1–5]. Mosquitoes of the *Aedes*, *Anopheles* and *Culex* genera are the primary vectors of *D*. *immitis*, which is transmitted when an infected mosquito carrying third-stage larvae (L3) feed on susceptible animals [1, 6]. The global prevalence of canine heartworm infection varies significantly throughout regions of the world due to a variety of different epidemiological factors, particularly a reliance of mosquito vectors on warm temperatures for survival [1, 3]. Within an Australian context, earlier research reported that canine heartworm infection is widespread and highly prevalent, particularly in areas of the states of Queensland (QLD), the Northern Territory and New South Wales (NSW) [5]. The current prevalence of infection in Australia is likely to have significantly reduced in recent years due to the intensive use of macrocyclic lactone (ML) preventatives, yet there is a paucity of prevalence data since the late 1990s [5]. Recent studies have sought to update the prevalence and distribution of *D*. *immitis*, with the results confirming the endemicity of canine heartworm in QLD [5, 7]. Despite these efforts, information on the occurrence of canine heartworm in reservoir populations, namely rescue and shelter dogs lacking preventative medication, remains scarce.

Diagnosis of canine heartworm infection and disease relies on the detection of *D*. *immitis* antigen in canine whole blood, serum or plasma and/or detection of *D*. *immitis* microfilariae (Mff) in whole blood [4, 8, 9]. Currently, enzyme-linked immunosorbent assays (ELISAs) are considered the most effective method of detection of *D*. *immitis* antigen [3, 9–11]. Heat treatment of samples prior to *D*. *immitis* antigen detection assays has been suggested to increase test sensitivity, as it has been shown to free bound antigens from immune complexes [4, 10–15]. However, heat treatment protocols contain multiple steps as well as chemicals to dissociate bound antigens, and have since been excluded from commonly used in-house test protocols in order to reduce operator error [3, 11, 13, 15–17]. More recently, studies have reported that heat treating samples on a dry heat block prior to antigen detection tends to improve detection of *D*. *immitis* antigen in a given sample, leading to a higher proportion of positive samples, or to increased optical density (OD) values of positive samples after heat treatment [2, 4, 10, 17–19]. Others have proposed a need for immune complex dissociation protocols to be further studied and improved prior to regular heat treatment use [13, 19, 20].

The aim of this study was to determine the prevalence of heartworm infection as assessed by the presence of canine heartworm antigen or Mff in stray and surrendered shelter dogs in QLD, Australia. Using blood samples collected from these dogs, we evaluated samples before and after heat treatment using a laboratory-based ELISA assay detecting *D*. *immitis* antigen. Antigen detection was coupled with a modified Knott’s test to detect the presence of Mff and with PCR for detection of *D*. *immitis* DNA. The results of this study enable recommendations for laboratory-based procedures for the detection of *D*. *immitis* antigen and demonstrate the latitudinal distribution of *D*. *immitis* in coastal areas of QLD, Australia.

## Methods

### Study design

**Fig. 1:**
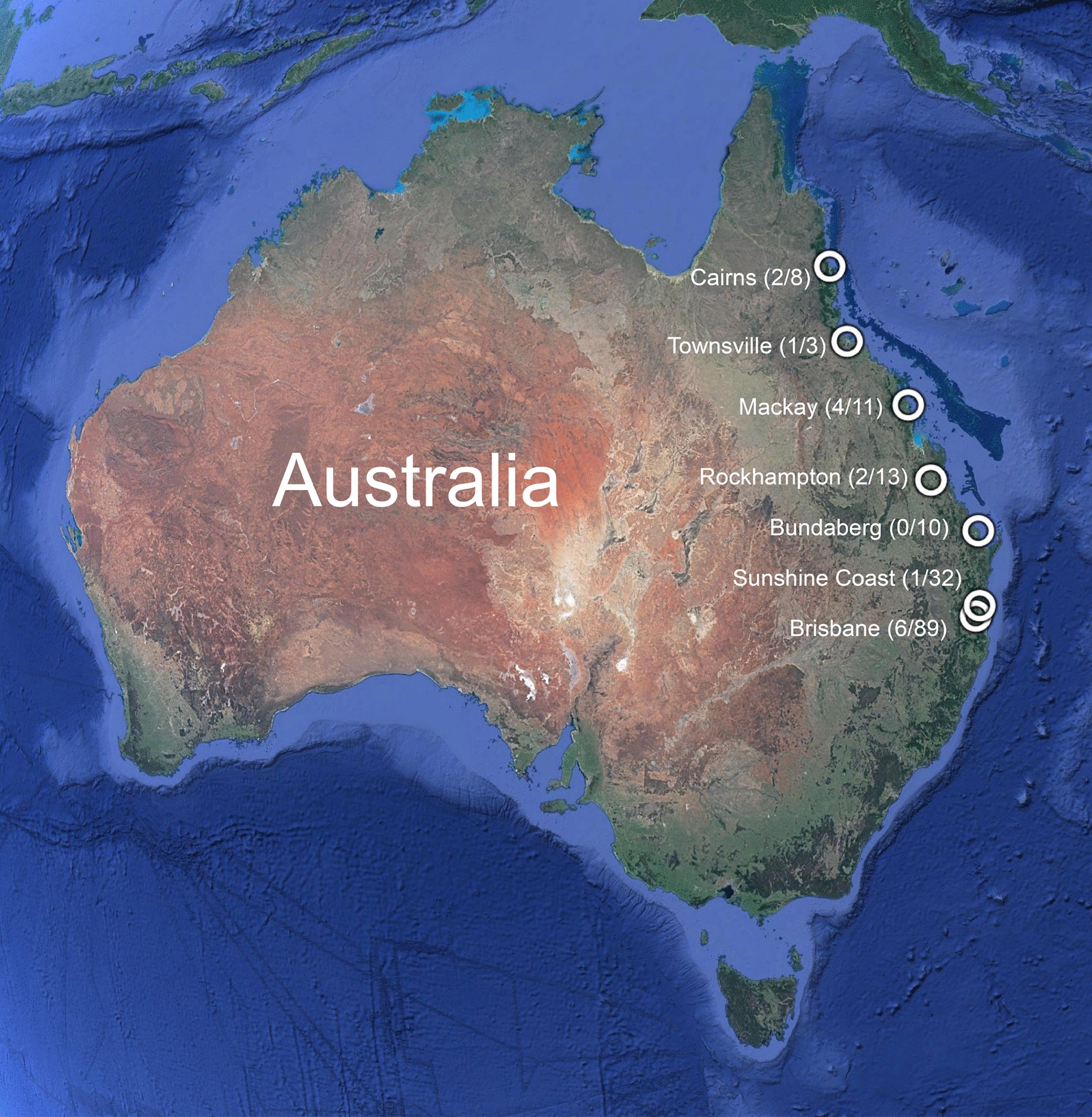
Location of shelters in Queensland, Australia accessed for the 2020 prevalence surveys where dogs were surveyed for the presence of *Dirofilaria immitis* antigen. The white circles indicate a location that is associated with the town name, the number in parentheses indicates the number of *D*. *immitis*-positive dogs using any test/total number of tested dogs. The shortest (airline) distance between Brisbane and Cairns is 1389.40 km. Image from is Google Earth; data are from SIO, NOAA, U.S Navy, NGA, GEBCO (Image Landsat/Copernicus)

Blood samples (*n* = 166) were collected directly from shelter dogs in QLD, Australia into EDTA blood collection tubes over a period of 3 months (January–March 2020) (Fig. 1). Dogs were housed in one of seven shelter locations (Brisbane, *n* = 89; Bundaberg, *n* = 10; Cairns, *n* = 8; Mackay, *n* = 11; Rockhampton, *n* = 13; Sunshine Coast, *n* = 32; Townsville, *n* = 3). Blood was collected from all dogs following concent from the shelter owners. Details of each dog (age, gender, source [stray or surrender]) were acquired immediately after blood collection. Blood samples were shipped on ice to the Veterinary Pathology Diagnostic Services (VPDS) laboratory, The University of Sydney, for processing. Whole blood was used immediately for modified Knott’s testing. Whole blood and plasma aliquots were stored at − 20 °C before further processing.

### Detection of Mff using the modified Knott’s test

Blood samples were immediately processed using a standard modified Knott’s test for the detection of Mff [8]. In brief, blood samples (1 ml) were combined with 2% buffered formalin (9 ml) in a 15-ml tube and gently homogenised. The homogenate was centrifuged (10,000 × *g*, 10 min) and the supernatant decanted. The resultant pellet was mixed with 2 drops of methylene blue, and enumeration of *D*. *immitis* Mff was performed under a light microscope at 10× magnification (Olympus Australia Pty Ltd., Notting Hill, VIC, Australia).

### Detection of *D*. *immitis* antigen before and after heat treatment

Plasma samples were tested for the presence of *D*. *immitis* antigen using the DiroCHEK Canine Heartworm Antigen Test Kit (DiroCHEK; Zoetis Australia Pty Ltd., Rhodes, NSW, Australia), which consists of a 96-well format ELISA, according to the manufacturer’s instructions. Prior to testing, stored samples were thawed at room temperature and then vortexed for 3 s. Unless sample volume was insufficient, 50 µl of unheated plasma was tested in duplicate from each sample (*n* = 165). Each testing batch included two positive and two negative control samples provided by the manufacturer.

Heat treatment was performed on plasma samples in duplicate where sufficient volume remained (*n* = 152) according to a previously described method [10, 17, 19]. Briefly, 70–200 µl of plasma was diluted 1:1 with phosphate-buffered saline (PBS; pH = 7.4) in a 1.5-ml plain tube and heated to 103 °C in a dry heat block for 10 min. Samples were then immediately placed on ice for 5 min before being centrifuged at > 10,000 ×  *g* for 5 min using a benchtop centrifuge (Eppendorf Australia, Eppendorf AG, Hamburg, Germany). The supernatant was collected and 50 µl was immediately tested using the DiroCHEK ELISA as described above.

A positive result was recorded if the sample developed a visible blue colour after 5 min of incubation at room temperature, as recommended by the manufacturer (Zoetis Australia). Immediately after samples were visually inspected, each plate was read on an ELISA reader (Halo LED96 Microplate Reader; Precisa Gravimetrics AG, Dietikon, Switzerland) using a 620-nm filter, and the OD for each sample was recorded. Samples were considered positive if the OD of the sample exceeded the mean negative control OD by more than three standard deviations.

### Molecular detection of *D*. *immitis* and filarial DNA

DNA was isolated from 200 µl of frozen whole blood samples by following the mammalian blood protocol of the Monarch Genomic DNA Purification Kit (New England Biolabs [Australia] Pty Ltd., Notting Hill, VIC, Australia) and eluted into 100 µl, before being stored at − 20 °C prior to processing. The presence of canine DNA was verified using a real-time PCR (qPCR) amplifying the partial canine glyceraldehyde-3-phosphate dehydrogenase (GAPDH; primer set RTPrimerDB ID: 1193), as previously described [5].

For species-specific detection of *D*. *immitis* we used a TaqMan probe assay with primers S0624 (F1: 5′-TAG AGG GTC AGC CTG AGT TAT C-3′) and S0626 (R1: 5′-AGT AGA ACG TAT ATT CTG AAC AGT AAC C-3′) and a TaqMan probe (S0625; 5′-FAM-AGA ACC AAT ACC AAC AGT ATG AAG ACC-BHQ1-3′). Reactions were run at a final volume of 20 μl, including 2 μl template DNA, 10 μl of *Sso*Advanced Universal Probes Supermix (BioRad Laboratories Pty Ltd., Gladesville, NSW, Australia) and primers and the probe at a final concentration of 400 and 100 nM, respectively. The PCR cycling conditions included an initial step at 95 °C, 3 min; followed by 40 two-step cycles of 95 °C/5 s and 60 °C/15 s. Each run included at least one negative (non-target control [NTC]) control and a blank DNA isolation control. A positive control DNA sample from a *D*. *immitis* adult was included to monitor for PCR inhibition. PCR testing was performed using a CFX96 Touch™ Real-Time PCR Detection System with the corresponding CFX Manager v.3.1 software (BioRad Laboratories Pty Ltd.). The qPCR threshold was determined automatically using default settings and threshold cycle (C_q_)-values were reported for each sample. Samples that amplified with a C_q_-value < 40 were considered positive.

To evaluate the presence of other arthropod-borne Mff, a high-resolution melt (HRM) real-time PCR assay was performed using primers designed by Wongkamchai et al. [21] as previously reported [7]. Positive controls included plasmid DNA for *D*. *immitis* and the *Acanthocheilonema reconditum *12S rRNA gene (GeneArt; Thermo Fisher Scientific Australia Pty Ltd., Scoresby, VIC, Australia). Each run included at least one negative control. Samples that amplified with a C_q_-value < 40 were sent for DNA sequencing using their respective amplification primers (Macrogen Inc., Seoul, Korea). Sequence chromatographs were manually inspected and compared to a reference *D*. *immitis* and *A*. *reconditium *12S rRNA gene sequences using CLC Main Workbench 6.9.1. (CLC Bio; Qiagen Pty Ltd., Chadstone, VIC, Australia).

### Statistical analysis

Samples were categorised into the following risk factors: age (≤ 2 years, > 2 years old), location (northern QLD [comprising Cairns, Mackay, Rockhampton and Townsville] or southern QLD [comprising Brisbane, Sunshine Coast and Bundaberg]), gender (both entire canine population and neutered males and females, respectively) and source (stray or surrender). Samples were assessed as antigen positive or negative for each of the different risk factors being assessed. Samples were excluded if there were insufficient plasma (< 50 µl) available to perform the ELISA assay (*n* = 1; one dog from Bundaberg).

Odds ratios (OR) for each of the different risk factors were calculated using binary logistic regression. A forwards stepwise logistic regression model was used to determine the best predictors of sample positivity, and the absence of confounding was verified (IBM SPSS v24; IBM Corp., Armonk, NY, USA). Cohen’s kappa coefficient (*κ*) was calculated between replicates to ensure reliability of testing (Microsoft Excel 2010; Microsoft Corp., Redmond, WA, USA). Test power was calculated using ‘Post-hoc Power Calculator’ (https://clincalc.com/stats).

True prevalence was calculated using previously published specificity and sensitivity values obtained from past studies [9, 22–25]. Sensitivity and specificity values were entered with total counts from this study into Epitools (Ausvet 2020; http://epitools.ausvet.com.au) to calculate true prevalence ranges.

## Results

### *Dirofilaria immitis* in surrendered and stray dogs from coastal Queensland

Samples from surrendered and stray dogs from coastal QLD (*n* = 166) were collected for the detection of *D*. *immitis* antigen. One blood sample had insufficient volume (< 0.5 ml; 20.01900-19; Bundaberg) and was excluded for antigen testing. A total of 7.9% (13/165; 95% confidence interval [CI]: 4.6–13.1%) unheated plasma samples tested positive for *D*. *immitis* antigen according to the DiroCHEK ELISA (Brisbane, 6/89; Sunshine Coast, 1/32; Bundaberg, 0/9; Mackay, 3/11; Rockhampton, 1/13; Townsville, 1/3; Cairns, 1/8). Duplicate testing on unheated plasma samples was performed on 95/165 samples, subject to sample availability (Cohen’s* κ* = 0.99).

The modified Knott’s test was performed on 161 blood samples (5 samples had insufficient blood volume [< 1 ml; 20.01847-11, 20.01900-9, 20.01900-10, 20.01900-19 and 20-01847-1]). Microfilariae of *D*. *immitis* were detected in eight blood samples (5.3%; 8/151; 95% CI: 2.6–10.3%; range: 2–3208 Mff/ml) from four different shelters (Brisbane, 3/89, Sunshine Coast, 1/32, Cairns, 1/7, Mackay, 3/10).

Canine DNA was successfully isolated from 166 blood samples, confirmed by the detection of canine DNA (average. C_q_-value: 20.0). DNA of *D*. *immitis* was detected in ten samples using a combination cytochrome* c* oxidase subunit 1 mitochondrial gene (*cox*1) and 12S rDNA real-time PCR assays (Additional file 1: Table S1). DNA of *D*. *immitis cox1* was detected using a real-time TaqMan PCR in 8/166 (4.8%; 95% CI: 2.3–9.4%) samples (C_q_-value range 21.2–30.6), all of which were Mff-positive according to the modified Knott’s test (Additional file 1: Table S1). Using an arthropod-borne HRM real-time PCR assay targeting 12S rDNA, all eight Mff-positive samples and a further two amicrofilaraemic samples were confirmed to contain *D*. *immitis* based on DNA sequences with 100% homology to reference 12S rDNA of *D*. *immitis*. The two amicrofilaraemic samples (20.01847-8, 20.01900-7; Additional file 1: Table S1) had high C_q_-values (37.3, 39.1) in the real-time PCR targeting 12S rDNA.

### Heat treatment increases the OD of the *D*. *immitis* antigen in positive samples

Heat treatment was performed on 152 canine plasma samples for which there was sufficient volume, including 54 duplicate samples. In 150/152 samples (Cohen’s* κ* = 0.98), heat-treated samples returned identical results to their unheated counterparts. In total, 8.5% of the submitted samples (14/165; 95% CI: 5.0–13.8%) from surrendered and stray dogs from QLD were positive for *D*. *immitis* antigen using either unheated plasma and/or heat-treated plasma.

Heat treatment did not significantly change the OD of all samples or antigen-negative samples for which both unheated and heat-treated ODs were available (Wilcoxon matched-pairs signed rank test, two-tailed, *P* = 0.09 and 0.66, respectively). Heat treatment, however, significantly increased the OD of the *D*. *immitis* antigen-positive samples (Wilcoxon matched-pairs signed rank test, two-tailed, *P* < 0.01) (Additional file 1: Table S1). The OD was increased by > 0.1 for 10/14 (71%) samples. Two samples had discordant results; the first sample (20.01900-7) was initially negative, but after heat treatment was considered to be antigen positive (Additional file 1: Table S1; note that this sample was PCR positive for *D*. *immitis* DNA); the second sample (20.01900-5) was initially considered to be antigen positive, but after heat treatment was considered to be negative and *D*. *immitis* DNA was not detected in the PCR analysis (Additional file 1: Table S1).

### Location is a significant risk factor for infection with *D*. *immitis*

Combining all tests (antigen tests with unheated plasma and heat-treated plasma, modified Knott’s tests and PCR) a total of 9.6% (16/166; 95% CI: 5.9–15.2%) of samples from surrendered and stray dogs from coastal QLD were positive for *D*. *immitis* (Additional file 1: Table S1).

Location was the only risk factor found to be significantly associated with whether a dog tested positive for *D*. *immitis* antigen (*P* = 0.01, binary logistic regression models). No other factors (age, gender or source) were significantly associated with the detection of canine heartworm (Table 1). Sequentially including age, gender and source in the model containing location did not substantially alter the estimated OR, and so none of these factors were considered to be confounders of the relationship between location and odds of testing positive. Dogs located in northern QLD were 4.39-fold (95% CI: 1.26–13.51) more likely to test *D*. *immitis* antigen positive than dogs located in southern QLD (Table 1). Including all *D*. *immitis* antigen- and/or Mff-positive samples (modified Knott’s test, PCR; see Additional file 1: Table S1) had no effect on the above result (OR: 6.13; 95% CI: 2.09–17.86). The tests probably had low power, however, for age (3.7% post-hoc power) and source (22% post-hoc power).

**Table 1:** Risk factors in dogs tested for *Dirofilaria immitis* antigen from Queensland, Australia

Risk factors	Positive for *D. immitis* antigen^a^	Negative for *D. immitis* antigen	Total	*P* value	Odds ratio	95% Confidence interval
Location^b^				0.010		
Southern QLD	7	123	130		1	−
Northern QLD	7	28	35		4.39	1.26–13.51
Sex/status				0.474		
Male neutered	3	64	67		1	−
Female entire	4	25	29		3.41	0.71–16.36
Female neutered	5	43	48		2.48	0.56–10.93
Male entire	2	19	21		2.25	0.35–14.44
Age				0.868		
≤ 2 years	7	72	79		1	−
> 2 years	7	79	86		0.91	0.31–2.73
Source				0.299		
Stray	10	125	135		1	−
Surrender	4	26	30		1.92	0.56–6.61
Total	14	151	165			

^a^Positive and negative counts are based on visual assessment of combined results for unheated and heat-treated plasma samples

^b^Northern Queensland (QLD) consists of samples obtained from Cairns, Mackay, Rockhampton and Townsville; and southern QLD consists of samples from Brisbane, Sunshine Coast and Bundaberg

Combining the results of the DiroCHEK ELISA on unheated and heat-treated plasma, samples collected from northern QLD revealed an apparent *D*. *immitis* prevalence of 20.0% (7 positive samples/35 tested) and an estimated true prevalence in the range of 8.0–29.1%, which was significantly higher than that of southern QLD where samples revealed an apparent *D*. *immitis* prevalence of 5.4% (7 positive/130 tested) and an estimated true prevalence in the range of 0.0–7.0% (Tables 1, 2).

**Table 2:** Apparent and true prevalence for canine heartworm antigen positivity of dogs from northern and southern Queensland, Australia

Location	Apparent prevalence (95% confidience interval,* n*)	True prevalence
Northern QLD	20.0% (9.7–36.2%, 35)	8.0–29.1%
Southern QLD	5.4% (2.4–10.9%, 130)	0.0–7.0%
Overall QLD	8.5% (5.0–13.8%, 165)	0.0–11.0%

The test results are based on combined unheated and heat-treated plasma testing. Sensitivity and specificity values used were chosen from previously published reports as follows: 0.88, 0.95; 0.77, 0.85; 0.90, 0.96; 0.86, 0.97; 0.77, 1; 0.85, 1; 0.71, 0.94; 0.63, 0.97 [9, 22–25]

## Discussion

### Location is a significant risk factor for canine heartworm infection

Our study demonstrate that the location of the animal (northern or southern QLD) poses a significant risk for the detection of *D*. *immitis* antigen in surrendered and stray dogs in coastal Queensland, Australia (Table 1). Early research has recognised that differences in the distribution of canine heartworm in Australia are likely due to the distribution of the multiple vector species [26, 27]. Mosquitoes of the *Aedes*, *Culex* and *Anopheles* genera have been demonstrated as the main vectors within Australia [28–31]. In order for transmission between the vector and the host to occur, the mosquito becomes infected through feeding on a microfilaraemic host, following which *D*. *immitis* matures from first-stage larva (L1) to L3 within the mosquito [3]. This maturation process is temperature dependent, with ideal conditions of 27 °C with 80% humidity allowing development within 10–14 days. The development process is more rapid under warmer conditions and slower under cooler conditions [3, 32, 33].

Multiple studies have demonstrated the influence of climate and climate change on the distribution of mosquito vectors, and thus the occurrence of canine heartworm infection [1, 7, 27, 32–35]. These implications are relevant to the current study as the average minimum–maximum temperature and humidity between 1999 and 2020 in Brisbane (southern QLD) was 16.4–26.6 °C and 52–63%, compared to an average temperature of 20.8–29.1 °C and 62–72% humidity in Cairns (northern QLD) [36, 37]. Climatic differences across QLD suggest that these variations are responsible for an increased number of mosquito vectors in northern QLD, and for a decreased maturation time of *D*. *immitis* within mosquitoes. Our results found that dogs residing in northern QLD are at greater risk of developing canine heartworm disease than dogs residing in southern QLD, which is supported by climatic data [36,37]. Further research is needed in this area to gain an accurate idea of the distribution of mosquito vectors across different regions of QLD, Australia.

### Prevalence of canine heartworm infection in Queensland, Australia

Canine heartworm infection has been reported in QLD with a prevalence between 11.8 and 36% [27, 38, 39]. These reports are largely out of date and are unlikely to reflect the current prevalence due to the uptake of heartworm preventatives in Australia [5, 40]. More recently, a 2016 paper confirmed the endemicity of canine heartworm in coastal areas of central QLD, Australia, despite a majority of the cohort being treated with monthly heartworm preventatives [7]. The current study demonstrates the prevalence of *D*. *immitis* antigen in 20.0 and 5.4% of surrendered and stray dogs from northern and southern QLD, respectively (Table 2). The ELISA antigen test used in this study performs well in detecting *D*. *immitis* infection in dogs with female worms, but it is well acknowledged that the sensitivity is lowered for infections from a single female or male-only infections [9, 22–25]. In a low burden cohort study of necropsy-confirmed positive dogs from the USA, the sensitivity and specificity of DiroCHEK ELISA was 94% (95% CI: 76–98%) and 94% (95% CI: 98–97%), respectively, for dogs with at least two adult female *D*. *immitis* [9]. In the same study, using all infected animals regardless of the burden, the sensitivity was 71% (95% CI: 62–79%) [9]. Similarly, the sensitivity and specificity of the DiroCHEK ELISA was 62.5 and 97.4%, respectively, using necropsy-confirmed positive dogs from Australia [23]. As our results are lower than historical prevalence estimates, they confirm the expectation that prevalence of the disease has decreased with the introduction of ML heartworm preventative medications [7, 26, 38, 39]. A limitation of this study is the incomplete or unknown history of canine heartworm prevention in the dogs tested herein, and hence the reported prevalence is potentially an underestimation of the true extent of *D*. *immitis* in QLD, Australia.

Donnett et al. [41] hypothesised that there was likely to be an increased prevalence of canine heartworm infection in shelter dogs compared to privately owned dogs due to increased preventative administration in the latter group. Based on the results of their study, these authors concluded that the presence of *D*. *immitis* was 34.4% higher in shelter dogs than owned dogs in Mississippi, USA, and suggested that these dogs could serve as reservoirs of heartworm infection for the rest of the country [41]. Similarly, in 2010 Tzipory et al. [42] found a significantly higher prevalence of *D*. *immitis* infections in shelter dogs (14.6%) compared to pet dogs (< 2%) [42]. Clearly, heartworm is still endemic in QLD, but the true prevalence in the owned dog population remains unknown, and implications of a high *D*. *immitis* prevalence within QLD shelter dogs on the wider owned dog population are also unknown. Future research is required to determine the number and distribution of owned dogs in QLD affected by canine heartworm infection, and to estimate the overall *D*. *immitis* prevalence.

Our results indicate that heat treatment did not have a significant effect on the detection of *D*. *immitis* antigen within canine plasma samples collected in QLD, Australia. In the past, studies explored the role of heat treatment in freeing *D*. *immitis* antigen, which is bound or trapped within immune complexes within canine plasma. Antigens freed from immune complexes resulted in an increased sensitivity of detection of *D*. *immitis* antigen post-heat treatment [2, 4, 10, 13, 17, 19, 43–45]. This theory of heat-induced immune complex destruction is based on earlier literature [11, 16]. Unfortunately, the protocols described in these studies are complex and require multiple steps and solutions. Further, Weil et al. [11] states that the success of their proposed assay was only in part due to pre-treatment with heat [11]. Therefore, findings in the recent literature about the impact of heat-treatment on assay sensitivity should be interpreted with caution. The American Heartworm Society (https://www.heartwormsociety.org/) [3] does not currently recommend heat treatment in conjunction with in-house antigen testing as it is unknown whether there may be cross-reactivity with other helminth antigens due to the heating of plasma samples. Antigen testing is now done with whole blood, sera and plasma, and it appears to be unknown if the heat treatment effect would be consistent across all three sample types. Instead, it is recommended that Mff testing be performed in combination with antigen testing [3]. The heat-treatment prior to *D*. *immitis* antigen testing may not be recommended for all dogs but may be beneficial for certain types of patients, as summarised in Table 5 in Little et al. [14]. Studies analysing known *D*. *immitis*-positive dogs, including exact worm burden and co-infection, will provide the needed insight into the value of heat treatment prior to testing for *D*. *immitis* antigen [13, 46].

The modified Knott’s test is traditionally used to detect Mff and, when coupled with microscopy, *D*. *immitis* can be reliably identified [8, 47, 48]. It is known, however, that a large proportion of heartworm-infected dogs are amicrofilaraemic [26]. For example, in QLD, Australia, Atwell et al. [49] detected Mff using the modified Knott’s test in 15/26 dogs confirmed to have patent infection at necropsy (35% amicrofilaraemic dogs) [49]. The DiroCHEK ELISA successfully identified 8/15 amicrofilaraemic dogs as *D*. *immitis* positive and the sensitivity for amicrofilaraemic dogs was estimated to be 53.3% (95% CI: 25.1–74.8%) [49]. An alternative approach to the concentration and visualisation of Mff using the modified Knott’s test is detecting *D*. *immitis* DNA in the blood samples.

There are several molecular tests that are either *D*. *immitis* specific or amplify conserved regions across other filarial nematodes [21, 50]. In this study, we showed that PCR reliably detected *D*. *immitis* in all Mff-positive samples, including those with as few as 2 Mff/ml. For PCR, we used only 0.1 ml of blood compared to the 1 ml required to conduct the modified Knott’s test demonstrating the usefulness of the former approach in low-volume samples. Successful amplification of samples with 2 Mff/ml is valuable because it suggests the presence of cell-free DNA (not confined to Mff) and that PCR can be used in low microfilaraemic samples (< 10 Mff/ml).

## Conclusions

In this study we confirmed the current presence of *D*. *immitis* in shelter dogs along the coastal region of QLD, Australia. Our results demonstrate that dogs located in northern QLD are at significantly higher risk of being *D*. *immitis* positive than dogs located in southern QLD. Heat treatment of canine plasma demonstrated no significant additional effect on whether samples were classified as *D*. *immitis* antigen positive or negative using the DiroCHEK ELISA. A combination of *D*. *immitis* antigen testing with the detection of Mff either using the modified Knott’s test or PCR is preferred to maximise detection of *D*. *immitis*-positive dogs.

## Supplementary Information

**Additional file 1**: **Table S1**. Summary of* Dirofilaria immitis*-positive diagnostics results for Queensland, Australia dogs from shelters.

## Data Availability

The raw data analysed in this article is available at LabArchives: http://dx.doi.org/10.25833/kvb5-2s19.

## Abbreviations

ELISA: Enzyme-linked immunosorbent assay
Mff: Microfilariae
ML: Macrocyclic lactone
NSW: New South Wales
PBS: Phosphate-buffered saline
QLD: Queensland
