# Supplementary material for: Multiple diagnostic tests demonstrate an increased risk of canine heartworm disease in northern Queensland, Australia
Source: Parasit Vectors. 2021 Aug 9;14:393. doi: 10.1186/s13071-021-04896-y (PMC8351338; doi:10.1186/s13071-021-04896-y)
Supplement: Supplementary file 1 — Additional file 1: Table S1. Summary of Dirofilaria immitis-positive diagnostics results for Queensland, Australia dogs from shelters. [file 13071_2021_4896_MOESM1_ESM.docx]

**Additional file 1: Table S1.** Summary of *Dirofilaria immitis* positive diagnostics results for Queensland, Australia dogs from shelters.

| Sample ID | Locality | Age (years) | Unheated result | OD ^a^ | Heat-treated result | OD ^a^ | Modified Knott's result | Mff/ml | C_q_-value (*cox1*) | C_q_-value (12S rDNA) | DNA sequence identification |
| --- | --- | --- | --- | --- | --- | --- | --- | --- | --- | --- | --- |
| 20.01847-1 | Cairns | 2.0 | Positive | 0.047/NA | Positive | 0.112/NA | NA | NA | >40 | >40/>40 | - |
| 20.02212-4 | Cairns | 3.0 | Negative | 0.064/NA | Negative | 0.063/0.050 | Positive | 2 | 30.11 | 29.19/28.78 | *Dirofilaria immitis* |
| 20.01847-7 | Townsville | 2.0 | Positive | 0.042/0.046 | Positive | 0.080/0.196 | Negative | Nil | >40 | >40/>40 | - |
| 20.01847-8 | Mackay | 7.5 | Positive | 0.078/0.045 | Positive | 0.181/0.364 | Negative | Nil | >40 | 37.33/>40 | *D. immitis* |
| 20.01847-13 | Mackay | 1.7 | Positive | 0.068/0.150 | Positive | 0.326/NA | Positive | 948 | 25.56 | 23.63/23.25 | *D. immitis* |
| 20.01847-15 | Mackay | 3.1 | Negative | 0.066/0.060 | NA | NA/NA | Positive | 28 | 28.59 | 27.59/26.82 | *D. immitis* |
| 20.01847-17 | Mackay | 1.1 | Positive | 0.031/0.039 | Positive | -0.026/NA | Positive | 2 | 28.74 | 27.37/27.18 | *D. immitis* |
| 20.01900-5 | Rockhampton | 3.0 | Positive | -0.003/0.018 | Negative | -0.029/NA | Negative | Nil | >40 | >40/>40 | - |
| 20.01900-7 | Rockhampton | 1.0 | Negative | 0.003/NA | Positive | 0.022/-0.015 | Negative | Nil | >40 | 39.08/>40 | *D. immitis* |
| 20.02216-4 | Sunshine Coast | 2.5 | Positive | 0.108/NA | Positive | 0.239/0.288 | Positive | 3208 | 22.20 | 22.11/21.49 | *D. immitis* |
| 20.02480-12 | Brisbane | 3.1 | Positive | 0.031/0.257 | Positive | 0.309/0.174 | Negative | Nil | >40 | >40/>40 | - |
| 20.02480-40 | Brisbane | 4.0 | Positive | -0.061/0.010 | Positive | 0.004/0.125 | Negative | Nil | >40 | >40/>40 | - |
| 20.03072-2 | Brisbane | 4.1 | Positive | 0.241/0.366 | Positive | 0.612/0.639 | Positive | 2600 | 21.23 | 20.55/20.07 | *D. immitis* |
| 20.03072-8 | Brisbane | 0.7 | Positive | 0.122/0.183 | Positive | 0.199/0.173 | Negative | Nil | >40 | >40/>40 | - |
| 20.03072-15 | Brisbane | 1.5 | Positive | 0.010/0.236 | Positive | 0.334/0.254 | Positive | 3040 | 23.75 | 22.88/22.13 | *D. immitis* |
| 20.03072-31 | Brisbane | 3.0 | Positive | 0.082/0.082 | Positive | 0.399/0.329 | Positive | 26 | 30.62 | 29.30/29.28 | *D. immitis* |

^a^ OD_620_ with subtracted mean+3SD of negative control.

Abbreviations: Mff, microfilariae; NA, sample not available.
